# Supplementary figures and images for: Improving Tirapazamine (TPZ) to Target and Eradicate Hypoxia Tumors by Gold Nanoparticle Carriers
Source: Pharmaceutics. 2022 Apr 12;14(4):847. doi: 10.3390/pharmaceutics14040847 (PMC9024542; doi:10.3390/pharmaceutics14040847)

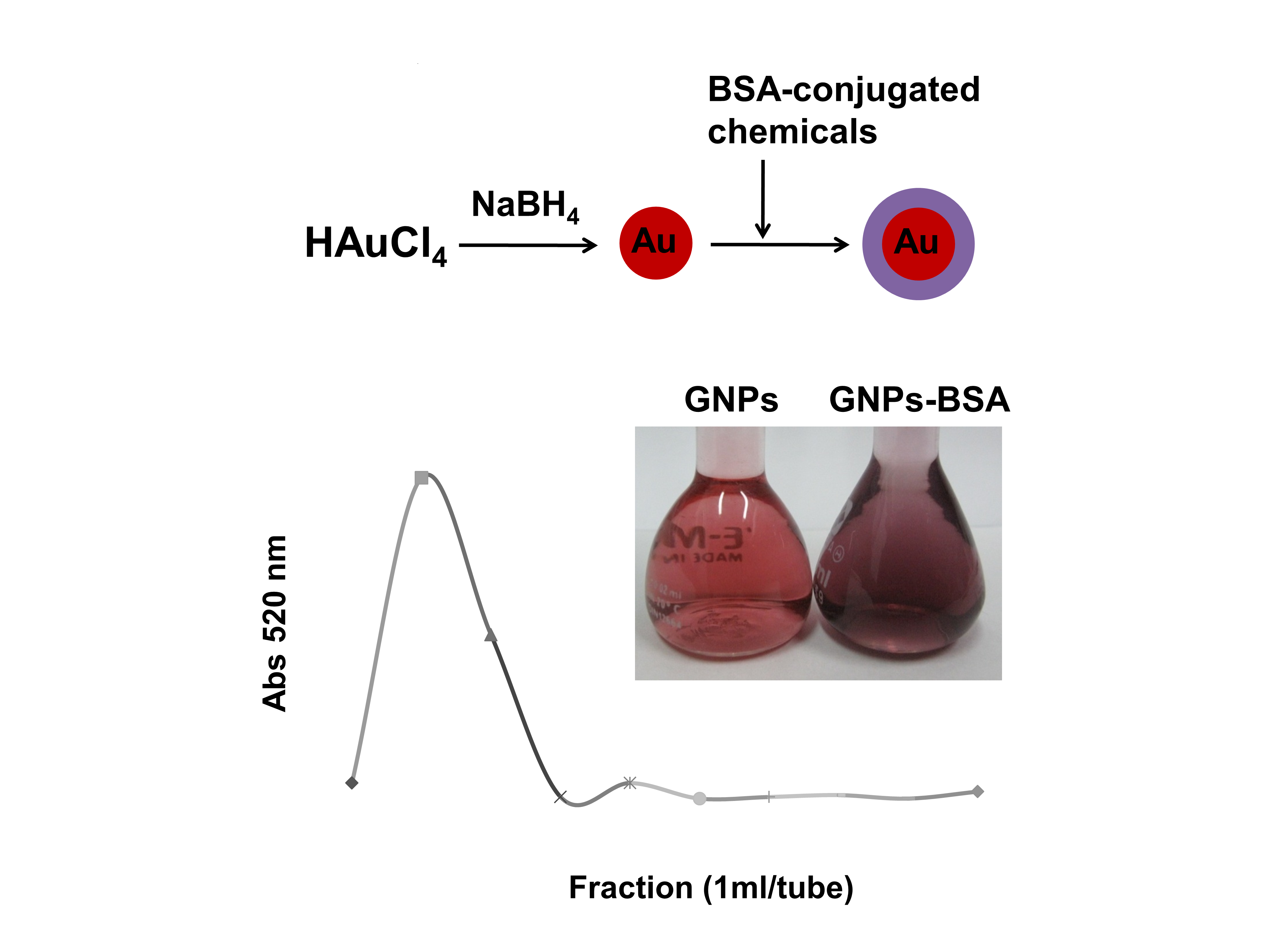

Supplement: Supplementary file 1 [file pharmaceutics-14-00847-s001.zip › Supplementary Figure S1.tif]

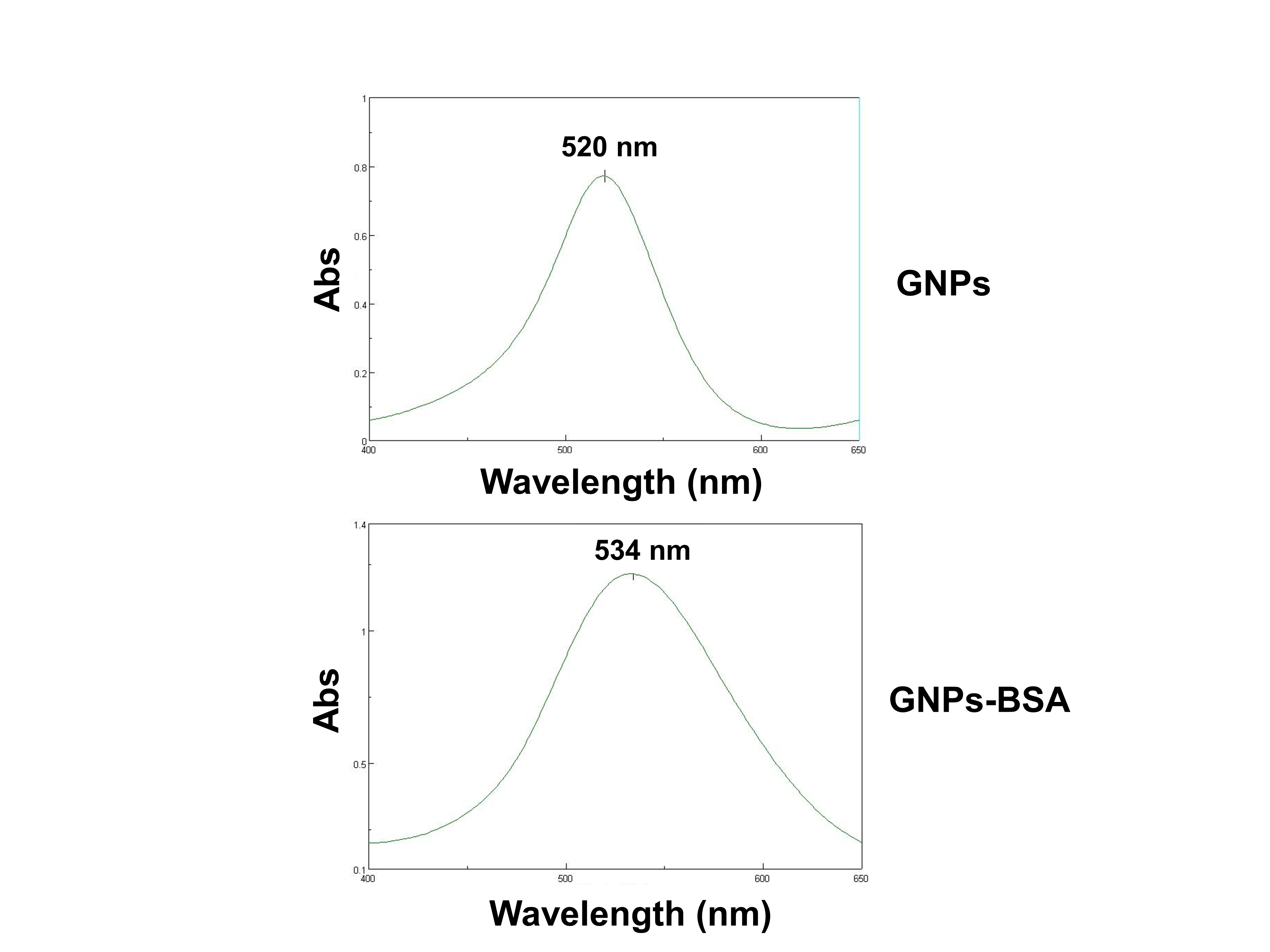

Supplement: Supplementary file 1 [file pharmaceutics-14-00847-s001.zip › Supplementary Figure S2.tif]
